# Metallocages for Metal Anions: Highly Charged [Co@Ge_9_]^5−^ and [Ru@Sn_9_]^6−^ Clusters Featuring Spherically Encapsulated Co^1−^ and Ru^2−^ Anions

**DOI:** 10.1002/anie.201907127

**Published:** 2019-08-21

**Authors:** Benedikt J. L. Witzel, Wilhelm Klein, Jasmin V. Dums, Marina Boyko, Thomas F. Fässler

**Affiliations:** ^1^ Department Chemie Technische Universität München Lichtenbergstrasse 4 85747 Garching Germany

**Keywords:** cobalt, intermetalloid cluster, Raman studies, ruthenium, tin

## Abstract

Endohedral clusters count as molecular models for intermetallic compounds—a class of compounds in which bonding principles are scarcely understood. Herein we report soluble cluster anions with the highest charges on a single cluster to date. The clusters reflect the close analogy between intermetalloid clusters and corresponding coordination polyhedra in intermetallic compounds. We now establish Raman spectroscopy as a reliable probe to assign for the first time the presence of discrete, endohedrally filled clusters in intermetallic phases. The ternary precursor alloys with nominal compositions “K_5_Co_1.2_Ge_9_” and “K_4_Ru_3_Sn_7_” exhibit characteristic bonding modes originating from metal atoms in the center of polyhedral clusters, thus revealing that filled clusters are present in these alloys. We report also on the structural characterization of [Co@Ge_9_]^5−^ (**1a**) and [Ru@Sn_9_]^6−^ (**2a**) obtained from solutions of the respective alloys.

The enormous structural diversity of intermetallic phases provokes the chemists’ imagination of an understanding of matter by manageable chemical concepts. The success of chemical concepts, such as valence, octet and (8‐N) rules, Lewis formulae and their extension to the VSEP concept, allow an understanding of the stability and the structure of most of the main‐group element molecules and have triggered many targeted syntheses of those. The progress of quantum‐chemical methods led to a deep understanding of these concepts. However, many more factors have to be considered for the description of intermetallic compounds. Whereas manageable concepts based on geometrical and electronical parameters, such as atomic size ratios (Laves^1^ and Frank‐Kasper^2^ phases) and valence electron concentration (Hume‐Rothery phases^3^) exist for a small group of intermetallic compounds, the rules developed by Zintl and their broader interpretation by Laves and Klemm^4^ facilitate the implementation of molecular concepts in intermetallic compounds. A formal electron transfer and salt‐like description allows the application of the 8‐N rule for the description of polymeric or oligomeric anionic substructures. An extension of this concept by including Wade's rules^5^ for polyhedral clusters has also been developed.^6^

Even though the simplicity of this borderline approach led to a broad acceptance of this concept, the extension to the large number of intermetallic compounds comprising transition‐metals is still missing. Nevertheless, some recent examples show that intermetalloid clusters, which are frequently used for the description of complex intermetallic compounds on a bare topological basis, similarly exist as discrete clusters and vice versa.^7^ The extension of Wade's rules originally developed for boranes to ligand‐free atom clusters arise from the fact that a certain number of energetically low‐lying orbitals exist, which are delocalized over all atoms. The same holds true for more complex cluster units, such as the nested onion‐like atom shells that occur as discrete [Sn@Cu_12_@Sn_20_]^12−^ units in the ternary phase Na_12_Cu_12_Sn_21_.^8^ Remarkably, structurally identical nested polyhedra Al@*M*_12_@Mg_20_ occur in Mg_32_(Al/Zn)_49_,^9^ with the difference that the outer atom shell is salt‐like in Na_12_[Sn@Cu_12_@Sn_20_], whereas it contains metallic bonds in *M*_12_[Al@*M*_12_@Mg_20_]. Owing to the very high negative charge of −12, an isolation of the large intermetalloid cluster [Sn@Cu_12_@Sn_20_] is not possible, however, the isoelectronic clusters [As@Ni_12_@As_20_]^3− [10]^ and [Sb@Pd_12_@Sb_20_]^3−/4− [11]^ have already been obtained from solution.

Endohedral molecular anions [*M*@*E_n_*]^*x*−^ with *M* being a transition metal, *E* a tetrel element, and *n=*9, 10, and 12 contain—as a result of the high coordination number of *M*—structural motifs as they occur in intermetallic compounds, nonetheless they allow a molecular description of chemical bonding. In this context ligand‐free endohedrally filled tetrel clusters with fully delocalized chemical bonds are interesting models for the understanding of chemical bonding in intermetallic compounds.^12^ For example, ^63^Cu NMR spectra revealed that the bronze‐type bimetallic clusters [Cu@Sn_9_]^3−^ and [Cu@Pb_9_]^3−^ are indeed [Cu^+^@(Sn_9_)^4−^] and [Cu^+^@(Pb_9_)^4−^] complexes with an unexpected positively charged Cu atom inside an electron‐rich and negatively charged p block metal cage.^13^ Tetrel element clusters can incorporate different metal atoms to give ligand‐free intermetalloid clusters, and numerous cage compounds have been obtained with different polyhedral shapes, which are obviously stabilized by the guest atom. Aside from the larger examples, such as twelve‐atomic entities, such as [Ru@Ge_12_]^3−^,^14^ or 17‐14f, 15 and 18‐atomic^16^ clusters with more than one guest atom, also ten‐14f, 17 and nine‐atomic clusters^13^, 15b, 15c, 16c, 18 of Ge, Sn, and Pb can host late transition metals (*TM*) as endohedral guest atoms. As a result of the stability of the filled d^10^ shell these endohedral guest atoms might function as a pseudo noble gas, and thus, for example, an endohedral Ni atom serves as a formal zero‐electron donor/acceptor,^19^ while a Co atom, for example, in [Co@Sn_9_]^5−^,15b, 18b is considered as a one‐electron acceptor site.^20^

The smallest intermetalloid deltahedral clusters to date are the nine‐atomic tetrel species, so far limited to the higher homologues Sn and Pb, with only one exception, namely the paramagnetic [Ni@Ge_9_]^3−^.^16^ The Co/Ge and Fe/Ge combinations are special cases, as the smallest clusters known so far, the [Co@Ge_10_]^3− [17]^ unit and the paramagnetic [Fe@Ge_10_]^3− [17c]^ anion, form pentagonal prisms without any deltahedral faces. Interestingly, quantum‐chemical calculations subsequently found this polyhedron as the structure of lowest energy for an arrangement of ten Ge atoms hosting an endohedral Co or Fe (or Pd, Pt) atom, while for the corresponding [Ni@Ge_10_]^2−^ and the bare [Ge_10_]^2−^ clusters a bicapped square antiprism is preferred,17b, 17c, 20, 21 and the size of the transition‐metal atom was considered as an influencing factor for the shape of the resulting polyhedron.^22^

In contrast to clusters in solution, much less is known about endohedrally filled tetrel‐atom [*E*_9_] clusters within intermetallic compounds and about the mechanisms how soluble clusters are formed from those.^23^ The only examples known are [Ni@Sn_9_] and [Co@Sn_9_] that occur in Na_12_NiSn_17_ and K_5−*x*_Co_1−*x*_Sn_9_ or K_13−*x*_Co_1−*x*_Sn_17_, respectively.15b, 18b Ternary alloys consisting of K‐Co‐Sn are suitable sources for the syntheses of intermetalloid clusters of higher nuclearity, such as [Co@Sn_9_Ni(CO)]^3−^, [Co@Sn_9_Pt(PPh_3_)]^3−^, or [Co@Sn_9_AuPh]^3−^.18c Examples of cobalt‐filled cages with negatively charged metal atoms exist also in mixed‐metal cages, such as [Co@Sn_6_Sb_6_]^3−^ and [Co_2_@Sn_5_Sb_7_]^3−^.^24^

Experimentally the formation of endohedrally filled clusters in solution seemed to be limited to clusters with a maximum charge of −4 as it is observed for bare [*E*_9_] clusters, whereas [Co@Sn_9_]^5−^ was solely detected in the solvent‐free solid.^25^

Herein we report on the syntheses of anionic clusters with even higher charges in solution, fathoming also the maximum size of an atom that can be incorporated as well as the charge transfer to the encapsulated transition‐metal atom.

[Co@Ge_9_]^5−^ (**1 a**) and [Ru@Sn_9_]^6−^ (**2 a**) are found in liquid ammonia solutions of melts of the nominal compositions “K_5_Co_1.2_Ge_9_” and “K_4_Ru_3_Sn_7_”, respectively. Compounds **1 a** and **2 a** are unusual examples with respect to the large size of the incorporated metal atoms (Ru), the stabilization of high negative charges of the transition metal (Ru^2−^), and the to date, highest charge of −6 for an endohedrally filled cluster. Quantum‐chemical analyses indicate the presence of [Co^1−^@(Ge_9_)^4−^] and [Ru^2−^@(Sn_9_)^4−^] cluster units.

Both compounds, K_6_(OH)[Co@Ge_9_]⋅16 NH_3_ (**1**) and K_7_(OH)[Ru@Sn_9_]⋅10 NH_3_ (**2**), were isolated as black single crystals from liquid ammonia solutions (experimental conditions are given in the Supporting Information). Owing to the presence of large amounts of ammonia solvent molecules in the lattice, the crystals are rather sensitive to temperature. Thus, all manipulations must be carried out at low temperatures. As a consequence of the experimental limitations arising when ammonia is used as a solvent and because of the low solubility of the highly charged clusters, a characterization of such clusters in solution is hindered. The good quality of the crystals obtained allowed for a detailed structural analysis by single‐crystal structure determination and comparison with theoretical results. The charge of the anion **1 a** is −5 and arises from the presence of six K^+^ cations and one hydroxide anion per formula unit.^26^ The crystallographic site symmetry of [Co@Ge_9_]^5−^ is *C_m_*, but the deviation from *D*_3*h*_ symmetry is small (Figure 1, Table 1). The point group of the [*E*_9_] clusters can generally be approximated by the geometric parameters given in Table 1.^27^ The structure of **1 a** is best described as a slightly distorted tri‐capped trigonal prism with almost equal prism heights and isosceles as well as nearly coplanar prism triangles. The Ge−Ge bond lengths are in the narrow range between 2.683 and 2.811 Å and thus within the range of binary Co−Ge phases (2.43–3.03 Å).^28^ All nine Co−Ge contacts are almost identical (2.331–2.374 Å) and are considerably shorter than those in the pentagonal prismatic [Co@Ge_10_]^3−^ cluster (2.48 and 2.53 Å) and slightly shorter than in Co−Ge phases (2.34 to 2.73 Å).^28^ In spite of such short Co−Ge distances, the incorporation of the Co atom into the [Ge_9_] cluster leads to a widening of the Ge−Ge bonds, and because of the uniformity of the Co−Ge distances, all the Ge cluster atoms are lying on an almost perfect sphere around the Co atom. Thus, the formation of a tri‐capped trigonal prism with *D*_3*h*_ symmetry is geometrically favored. For clusters with 22 skeleton electrons (SE), this polyhedron type is rarely observed, and for bare metals clusters lies energetically close to a mono‐capped square antiprism with *C*_4*v*_ symmetry.18b, 27 This shape thus indicates a 22 SE cluster and an incorporation of an atom with a d^10^ valence electron configuration and a formal charge distribution [Co^1−^@(Ge_9_)^4−^]. Anionic Co is known from several ligand‐stabilized complexes^29^ and has recently also been found as a guest ion incorporated in [Sn_9_] Zintl clusters.15b, 15c, 18b, 18c

**Figure 1:**
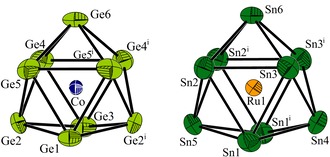
The endohedral clusters **1 a** and **2 a‐I** (**2 a‐II** is found in Figure S1). Displacement ellipsoids are set at the 80 % probability level; symmetry operation: (i) *x*, 0.5−*y*, *z*. Detailed bond lengths can be found in the Supporting Information.

**Table 1:** Geometrical parameters for the *TM*‐centered [*E*_9_] (*E*=Ge, Sn, and Pb) clusters in **1** and **2**, [Cu@Sn_9_]^3−^ and [Cu@Pb_9_]^3−^,^13^ and the corresponding values of their empty equivalents in K_5_(OH)[*E*_9_]⋅11 NH_3_ (*E*=Ge (**3**),^30^ Sn (**4**)^31^) and Li_4_Pb_9_⋅17 NH_3_.^34^ For a detailed explanation of the geometrical parameters see elsewhere.^32^ 
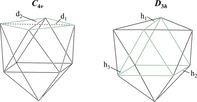

Compound	SE^[a]^	*h*_1_^[b]^	*h*_2_^[b]^	*h*_3_^[b]^	*d*_1_/ *d*_2_^[c]^	Point group	Volume/ Å^3[33]^	Volume change
[Ge_9_]^4−^ **3 a** 30	22	1	1	1.26	1.03	ca. *C*_4*v*_	21.3	–
[Co@Ge_9_]^5−^ (**1 a**)	22	1.22	1.22	1.25	1.12	ca. *D*_3*h*_	26.4	**19.4 %**
[Sn_9_]^4−^ **4 a** 34	22	1	1.01	1.35	1.03	ca. *C*_4*v*_	32.9	–
[Ru@Sn_9_]^6−^ (**2 a‐I**)	22	1.31	1.29	1.30	1.12	ca. *D*_3*h*_	38.3	**16.4 %**
[Ru@Sn_9_]^6−^ (**2 a‐II**)	22	1.28	1.28	1.26	1.17	ca. *D*_3*h*_	38.8	**17.9 %**
[Cu@Sn_9_]^3−**[13]**^	22	1.20	1.22	1.25	1.17	ca. *D*_3*h*_	37.3	13.4 %
[Pb_9_]^4−**[34]**^	22	1	1.01	1.28	1.03	ca. *C*_4*v*_	37.4	–
[Cu@Pb_9_]^3−**[13]**^	22	1.15	1.18	1.22	1.17	ca. *D*_3*h*_	41.7	11.3 %

[a] Number of skeleton electrons (SE) assuming a d^10^ configuration for the central atoms. [b] Heights of an underlying tri‐capped trigonal prisms (ca. *D*_3*h*_), values are normalized to the shortest height of the prism of each tetrel element cluster. [c] Ratio of diagonals of the planar square of an underlying mono‐capped square antiprism (ca. *C*_4*v*_).^35^

In K_7_(OH)[Ru@Sn_9_]⋅10 NH_3_ (**2**) the charge of **2 a** is −6, derived from the presence of seven K^+^ ions and one (OH)^−^ anion. Crystallographically, the 10 NH_3_ molecules per formula unit can clearly be distinguished from the OH group. In the unit cell there are two symmetrically independent clusters **2 a‐I** and **2 a‐II** which have the same shape. The analyses of the geometrical parameters (Table 1) again show polyhedra close to perfect *D*_3*h*_ symmetry with nine almost equal Ru−Sn bond lengths in the narrow range 2.641–2.700 Å and which are in good agreement with the corresponding values in the respective binary system (2.60–2.80 Å).^36^ The Sn−Sn distances (3.0512(8)–3.1409(6) Å) are in a narrow range and on average considerably longer than those in empty [Sn_9_]^4−^ (typically below 3 Å) but within the range of 2.86–3.64 Å observed in binary Ru−Sn compounds. Assuming an [Sn_9_]^4−^ host polyhedron with an elongated trigonal‐prismatic structure,^35^ the endohedral Ru atom must also provide 10 electrons to the system, resulting in a formally twofold negatively charge for Ru. Compounds with Ru(−II) are rare, but have been found in isonitrile^37^ and biphosphinine^38^ complexes, whereas to the only known intermetalloid cluster, [Ru@Ge_12_]^3−^ was attributed Ru(−I).14c

An inspection of other [Sn_9_] units reveals different structures for the empty, the Co‐filled, and the Ni‐filled 22‐electron clusters, namely *C*_4*v*_ for [Sn_9_]^4−^,^31^ [Ni@Sn_9_]^4−^,18a, 18b and for the partially oxidized [Co_0.68_@Sn_9_]^4−^ cluster with 21 SE,15c while [Cu@Sn_9_]^3−^ shows *D*_3*h*_ symmetry.^13^ Generally, in all cases of isolated [Sn_9_] units the distances between the transition metal and the capping Sn atom are significantly longer than the remaining *TM*–Sn bonds.

Compared to related cluster units with the same endohedral transition metal, **1 a** and **2 a** display in average much shorter *TM*–*E* bond lengths in a smaller range (see Supporting Information, Table S5; a full list of *E‐*‐*E* and *TM*–*E* bond lengths can be found in Tables S2 and S3). As a result of the presence of the endohedral atoms the *E*–*E* distances in **1 a** and **2 a‐I/II** are about 5 % longer than in the empty cluster analogues in **3** and **4**, respectively, which leads to an increase of the cluster volume of 19.4 % for **1 a** and 17.15 % for **2 a‐I/II** (see Table S4).

DFT calculations have been performed for **1 a** and **2 a** as well as for the empty [Ge_9_]^4−^ and [Sn_9_]^4−^ ions using a polarized continuum model (PCM) solvate field based on water for negative charge compensation. In accordance to experimental findings the structure optimization leads to a *D*_3*h*_ symmetry for **1 a** and **2 a** and to *C*_4*v*_ symmetry for the empty clusters. Compounds **1 a** and **2 a** represent minima with considerably large HOMO–LUMO gaps of 3.73 eV and 2.98 eV, respectively. The calculated interatomic distances agree well with the experimental data showing deviations of less than 0.06 Å or below 2 % for all Ge−Ge/Sn−Sn and Ge−Co/Sn−Ru distances for both endohedral clusters, which confirms the optimized structure. Charge distribution based on population analyses according to Hirshfeld and natural population analyses were carried out (all charges are given in the Supporting Information in Table S6), and both methods account for negative charges for the endohedral *TM* atoms.

To derive an orbital interaction diagram, single‐point calculations for the empty clusters [Ge_9_]^4−^ and [Sn_9_]^4−^ were performed in *D*_3*h*_ symmetry, resulting in slightly different HOMO–LUMO gaps if compared to the *C*_4*v*_ minimum structure (Tables S7 and S8).^39^

The molecular orbitals shown in Figure 2 b allow for a classification according to the tensor surface harmonic theory in S, P, D, F, and G orbitals.^40^ The 1S^2^1P^6^1D^10^2S^2^1F^14^2P^6^ electronic configuration with an empty 1G orbital as the LUMO perfectly fits the superatom model of the 40 electron clusters [*E*_9_]^4−^. The d orbitals of the Co^−^ or Ru^2−^ interact with a D set of the cluster representing delocalized bonds, whereas the F and G type orbitals are mainly unaffected. The alternative analysis by intrinsic bond orbitals^41^ (Figure 2 c, and Tables S9 and S10, Figures S7 and S8) shows that all interactions between the *TM* and the cluster atoms correspond to fully delocalized multi‐center bonds.

**Figure 2:**
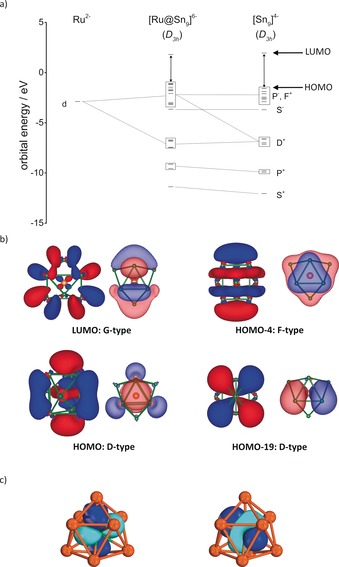
a) Molecular orbital interaction diagram of [Ru@Sn_9_]^6−^ in fragments of formal Ru^2−^ and [Sn_9_]^4−^ (*D*_3*h*_ symmetry). Important interactions are indicated by dashed lines. b) Selected molecular orbitals of [Ru@Sn_9_]^6−^ are represented in two orientations with iso‐values 0.02. According to their symmetry the orbitals are D, F, and G type. The HOMO corresponds to an anti‐bonding interaction of the d${}_{z{}^{2}}$ of the central atom with the D shell of the spherical cluster. c) Two representative [Ru@Sn_9_]^6−^ Intrinsic bond orbitals indicating multi‐center bonds.

The formation of such clusters, which leads to the anticipation of several mechanisms on a theoretical level is most intriguing, however, experimental data are scarcely reported.7a, 42 As stated above, generally two possible ways for the formation of endohedral clusters have to be considered: either they are formed during a solid‐state reaction or by an intermediate cluster opening step in solution. The powder X‐ray diffractograms of the solid precursors “K_5_Co_1.2_Ge_9_” and “K_4_Ru_3_Sn_7_” are rather complex but show the finger‐print characteristics of the K_13_CoSn_17_ structure which contains filled clusters (Figure S9). To detect such filled clusters in alloys, which are solely characterized by a crude phase assignment, we applied Raman spectroscopy for the first time.^43^ Owing to the absence of experimental data of such alloys, Raman frequencies calculated from the molecular anions [Co@Ge_9_]^5−^ and [Ru@Sn_9_]^6−^ were used as a reference. In case of the empty clusters, a close accordance with an acceptable error of 5 %^44^ is observed between the measured^45^ and the simulated data (Tables S6, S8). Even though more complex phonon modes must be considered in the solid, the strongest modes match very well the experimental data for both binary Zintl compounds K_4_Ge_9_ and K_4_Sn_9_ and may serve as a characteristic marker for the presence of empty [*E*_9_] clusters. The main mode of the [*E*_9_] cluster is a breathing motion, that is, inflating and deflating, which is observed at 220 cm^−1^ for [Ge_9_] and 146 cm^−1^ for [Sn_9_]. According to DFT calculations these modes are shifted to higher wavenumbers by about 25 cm^−1^ for [Co@Ge_9_]^5−^ and 40 cm^−1^ for [Ru@Sn_9_]^6−^, if the endohedral species is present, as shown in Figure 3 for [Co@Ge_9_]^5−^ (for [Ru@Sn_9_]^6−^ see Figure S11). The characteristic modes of filled clusters, however, are those which include the central atom. The vibrations of the discrete cluster anions **1 a** and **2 a** correspond to the modes **A** and **B** in the regions 360 and 260 cm^−1^, respectively, as shown for **1 a** in Figure 3. Fortunately these vibrations appear in a region where none of the related compounds shows any other modes,45a, 46 and thus it can be considered as an evidence for the presence of the filled clusters in the solid‐state precursors.

**Figure 3:**
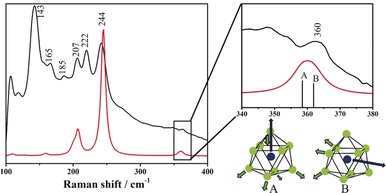
The Raman spectrum of the precursor “K_5_Co_1.2_Ge_9_” (black lines) and the calculated Raman spectrum of [Co@Ge_9_]^5−^ (red lines); the enlarged section shows the resonances of the vibration modes “**A**” and “**B**”.

The [Co@Ge_9_]^5−^ and [Ru@Sn_9_]^6−^ units are the endohedral clusters with the highest volume increase to date of filled clusters as compared to the corresponding empty ones. The synthesis proceeded from the elements via a two‐step route, first a solid‐state reaction, then subsequent dissolution of the precursor phase obtained in liquid ammonia. Additionally we established for the first time that Raman spectroscopy is a reliable probe to assign the presence of endohedrally filled clusters in intermetallic phases.

## Conflict of interest

The authors declare no conflict of interest.

## Supporting information

As a service to our authors and readers, this journal provides supporting information supplied by the authors. Such materials are peer reviewed and may be re‐organized for online delivery, but are not copy‐edited or typeset. Technical support issues arising from supporting information (other than missing files) should be addressed to the authors.

Supplementary
