# Supplementary material for: Metallocages for Metal Anions: Highly Charged [Co@Ge9]5− and [Ru@Sn9]6− Clusters Featuring Spherically Encapsulated Co1− and Ru2− Anions
Source: Angew Chem Int Ed Engl. 2019 Aug 21;58(37):12908–13. doi: 10.1002/anie.201907127 (PMC6771791; doi:10.1002/anie.201907127)
Supplement: Supplementary file 1 — Supplementary [file ANIE-58-12908-s001.pdf]

Angewandte Chemie
Eine Zeitschrift der Gesellschaft Deutscher Chemiker

Supporting Information

Metallocages for Metal Anions: Highly Charged $[Co@Ge_9]^{5-}$ and $[Ru@Sn_9]^{6-}$ Clusters Featuring Spherically Encapsulated Co^{1-} and Ru^{2-} Anions

*Benedikt J. L. Witzel, Wilhelm Klein, Jasmin V. Dums, Marina Boyko, and Thomas F. Fässler**

anie_201907127_sm_miscellaneous_information.pdf

1. Syntheses 2
2. Crystal structure determination 3
3. Raman spectroscopy 17
4. Computational Analysis 10
5. X-Ray powder diffraction (PXRD) 16
6. Differential Scanning Calorimetry (DSC) 20

1. Syntheses

General. All manipulations were carried out under argon atmosphere inside a glove box or by using standard Schlenk techniques. Flasks were dried at 650 °C *in vacuo* prior to use. Commercially available chemicals were used without further purification unless stated otherwise. Liquid ammonia is stored in a cooling trap over Na and evaporated into the reaction vessel as needed. 18-Crown-6 is sublimed at 80 °C and dynamic vacuum before usage. Cryptand[2.2.2] is dried overnight *in vacuo*.

$K_5Co_{1.2}Ge_9$. A phase with the nominal composition CoGe was synthesized from the elements (Co: 294.7 mg, 5 mmol; Alfa Aesar, 99.8 %; Ge: 363.3 mg, 5 mmol, Evochem, 99.999 %) using an arc melting furnace. The resulting regulus, composed of CoGe, Co_2Ge and $CoGe_2$ according to X-ray powder diffraction, was ground and mixed with 2.36 g Ge (32.5 mmol) and 814 mg K (20.8333 mmol; Merck, >98 %) to yield the molar ratio of $K_5Co_{1.2}Ge_9$. The mixture was sealed in a tantalum crucible, placed in an evacuated quartz glass tube and heated to 1000 °C with a rate of 2 °C/min, using a tube resistance furnace. The temperature was kept for 8 h, then lowered to 550 °C at a rate of 0.1 °C/min, kept there for 24 h, and finally the tube was quenched to room temperature on air.

$K_6[Co@Ge_9](OH) \cdot 16\ NH_3$ (1). 100 mg “$K_5Co_{1.2}Ge_9$“ (0.11 mmol) and 74.5 mg cryptand[2.2.2] (0.198 mmol) are dissolved in approx. 5 mL liquid ammonia. The resulting dark red solution is stored at -42 °C. After six months black crystals were isolated with a yield of about ca. 25 %, estimated from the total expected amount. So far, no by-products, e.g. cryptand-containing species, have been identified.

$K_4Ru_3Sn_7$. A phase with the nominal composition “Ru_3Sn_7“ was synthesized from the elements Ru (117.5 mg, 1.16 mmol; Alfa Aesar, 99.9 %) and Sn (321.9 mg, 2.71 mmol; Chempur, 99.999 %) using an arc melting furnace. The resulting regulus, composed of Ru_3Sn_7 and Ru_2Sn_3 according to powder X-ray diffraction, was ground and mixed with elemental K (60.6 mg, 1.55 mmol) to yield a molar ratio of $K_4Ru_3Sn_7$. The mixture was sealed in a tantalum crucible, placed in an evacuated quartz glass tube and subsequently heated to 1000 °C at a rate of 6 °C/min and held for 6 h. The temperature was lowered to 600 °C at 0.1 °C/min, held for 120 h and then cooled to room temperature at 1 °C/min.

$[K_7(OH)]RuSn_9 \cdot 10\ NH_3$ (2). 0.075 g “$K_4Ru_3Sn_7$” (0.058 mmol) and 0.027 g 18-crown-6 (0.105 mmol) were dissolved in approx. 5 mL of liquid ammonia and stored at -42 °C. After 2 months black crystals could be isolated with a yield of 25 %.

2. Crystal structure determination

The air- and moisture-sensitive crystals were transferred into perfluoropolyalkyl ether (Galden Perfluorniated Fluid LSD 230, *Solvay Speciality Polymers*) cooled with a stream of liquid nitrogen, and the selected single crystals were fixed into a nylon loop by the enclosing frozen ether. Data collections were carried out at 150 K on a STOE StadiVari diffractometer (Mo K_{α} radiation) equipped with a Dectris Pilatus 300K detector. The crystal structures were solved by Direct Methods (SHELXS97) and refined by full-matrix least-squares calculations against F^2 (SHELXL-2014).[1] Cluster volumes are calculated with VESTA.[2] During the structure refinements, it turned out that one additional anion per formula unit was required for charge balance. After closer inspection of the crystal structures, one light atom site differed strikingly from the remaining ammonia molecules with respect to its very small displacement parameter. Thus, this atom had to be assigned as oxygen, representing a hydroxide anion instead of an amide owing to significantly lower residual quality factors and physically senseful anisotropic displacement parameters after a refinement of oxygen compared to nitrogen at the respective atom site for both crystal structures. All non-hydrogen atoms were refined with anisotropic displacement parameters, and the hydrogen atoms were placed in calculated positions and refined by using a riding model. In compound **2**, one of the independent cluster units was found to be partially disordered in two superposing orientations, the minor individual (atoms Sn7B, Sn8B, and Sn9B) was refined to an occupation of 6.1(2) %. Further details have been deposited and may be obtained from FIZ Karlsruhe, 76344 Eggenstein-Leopoldshafen, Germany (fax: (+49)7247-808-666; e-mail: crysdata@fiz-karlsruhe.de), on quoting the deposition number CSD-1866534 ($K_6[Co@Ge_9](OH) \cdot 16\ NH_3$ (**1**)) and CSD-1866535 ($[K_7(OH)]RuSn_9 \cdot 10\ NH_3$ (**2**)).

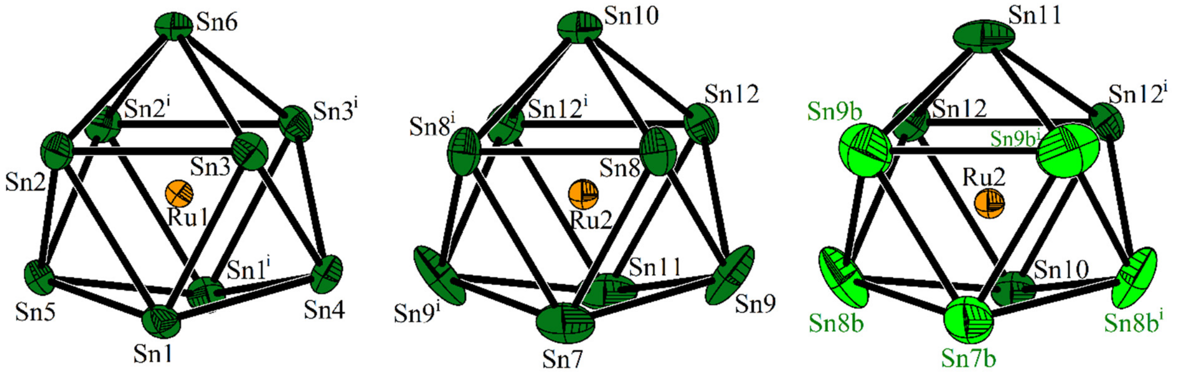

Figure S1. The crystallographically independent cluster units of compound **2**, **2a-I** (left) and the two orientationally disordered individuals of **2a-II** with atomic site occupations of 93.9(2) % (middle) and 6.1(2) % (right; light green atoms represent the disordered part with lower occupation); displacement ellipsoids are drawn at a 50 % probability level; symmetry operation: (i) *x*, 0.5-*y*, *z*.

Table S1. Crystallographic data and refinement details of the structure determination of $K_6[OH][Co@Ge_9] \cdot 16\ NH_3$ (**1**) and $K_7[OH][Ru@Sn_9] \cdot 10\ NH_3$ (**2**).

	$K_6[OH][Co@Ge_9] \cdot 16\ NH_3$ **(1)**	$K_7[OH][Ru@Sn_9] \cdot 10\ NH_3$ **(2)**
empirical formula	$CoGe_9H_{49}K_6N_{16}O$	$K_{14}O_2Ru_2Sn_{18}N_{20}H_{62}$
molecular weight [g/mol]	1236.39	3260.65
crystal system	orthorhombic	orthorhombic
space group	*Pnma* (no. 62)	*Pnma* (no. 62)
a [Å]	25.463(2)	36.970(7)
b [Å]	15.7469(12)	19.700(4)
c [Å]	10.0509(11)	10.370(2)
V [Å^3]	4030.1(6)	7553(3)
Z	4	4
T [K]	150(2)	150(2)
μ [mm^{-1}]	7.653	7.015
measured reflections	71159	197829
hkl ranges	$-31 < h < 31$<br>$-19 < k < 19$<br>$-12 < l < 12$	$-45 < h < 45$<br>$-24 < k < 24$<br>$-12 < l < 12$
$2\,\theta$ range	4.36 - 52.0	4.08 - 52.0
R_{int}	0.1584	0.1102
independent reflections	4111	7653
reflections with $I > 2\,\sigma$	2992	6025
parameters	170	277
R_1 (all; $I > 2\,\sigma$)	0.0425; 0.0675	0.0547; 0.0403
wR_2 (all; $I > 2\,\sigma$)	0.0978; 0.1124	0.1110; 0.1025
GooF	1.040	1.038
max. / min. residual electron density [e/Å^3]	1.13 / -1.11	4.23 / -2.11
depository no.	CSD-1866534	CSD-1866535

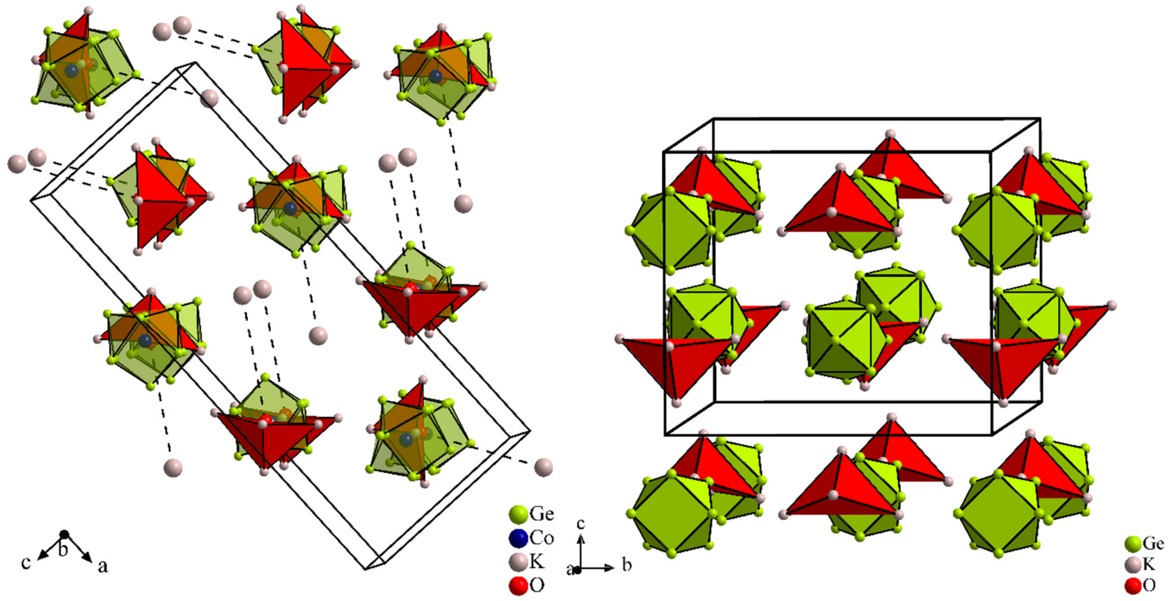

Figure S2. Left: Distorted, rock salt-related packing of $[K_6(OH)]^{5+}$ groups (red pyramids) and $[Co@Ge_9]^{5-}$ clusters (green centered polyhedra) in $K_6(OH)[Co@Ge_9] \cdot 16\ NH_3$. N and H atoms are omitted for clarity. The K5 atoms, which are not directly coordinated to the O atom, are connected by a dashed line to the pyramid illustrating the reason for the heavy distortion with respect to the more regular arrangement in $K_5(OH)[Ge_9] \cdot 11\ NH_3$ (right).[3]

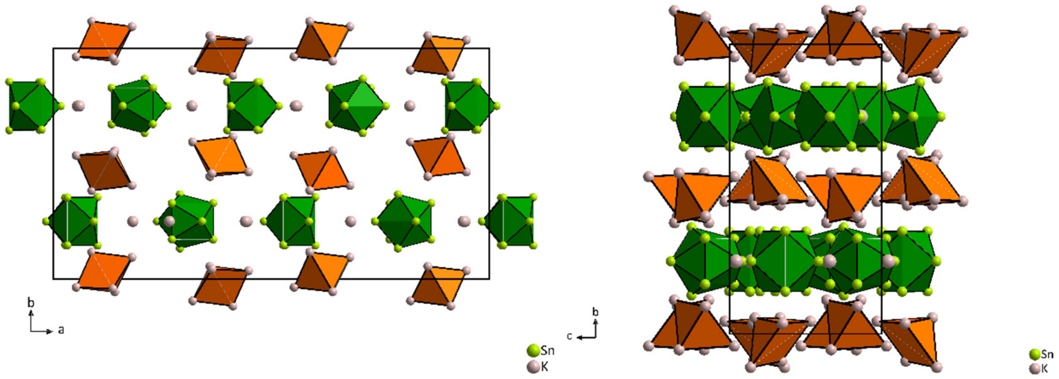

Figure S3. Layered packing of $[K_6(OH)]^{5+}$ groups (orange pyramids) and $[Ru@Sn_9]^{6-}$ clusters (green polyhedra) in $K_7(OH)[Ru@Sn_9] \cdot 10\ NH_3$ parallel to the *ac* plane. K atoms which are not coordinated to the O atoms, are drawn as grey spheres, N and H atoms are omitted for clarity.

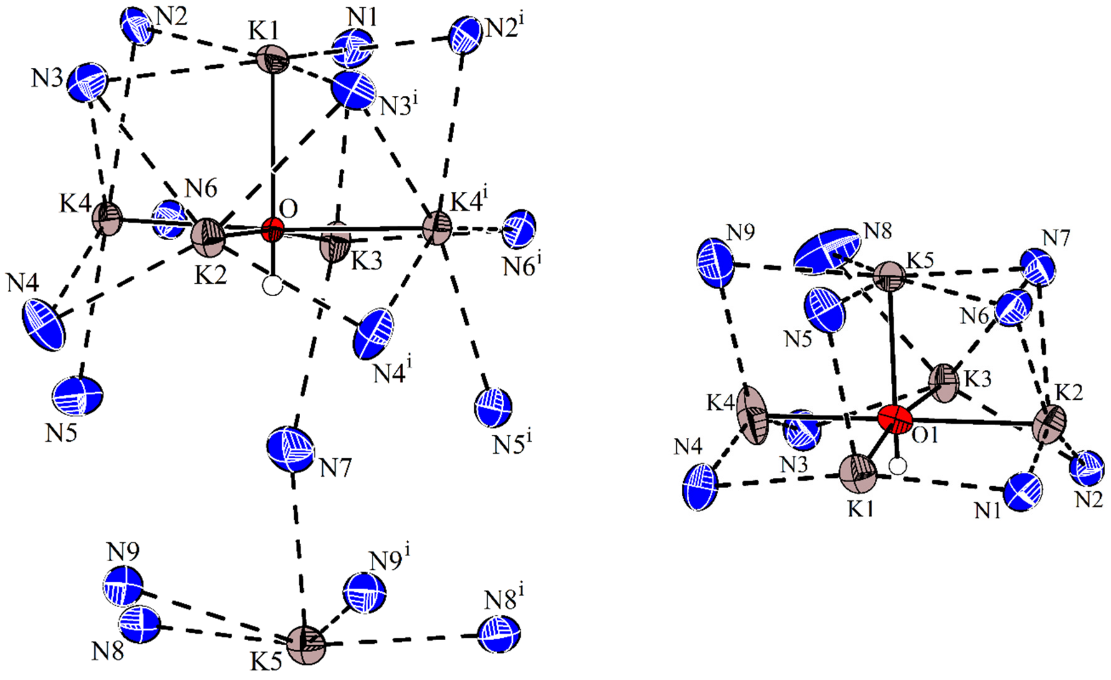

Figure S4. Pyramidal [K_5OH] units from **1** (left) and **2** (right); displacement ellipsoids are drawn at a 50 % probability level. Interatomic K-O distances below 2.7 Å are shown as solid lines, K-N distances as dashed lines; H atoms of the NH_3 molecules are omitted for clarity. Symmetry operation: (i) *x*, 0.5-*y*, *z*.

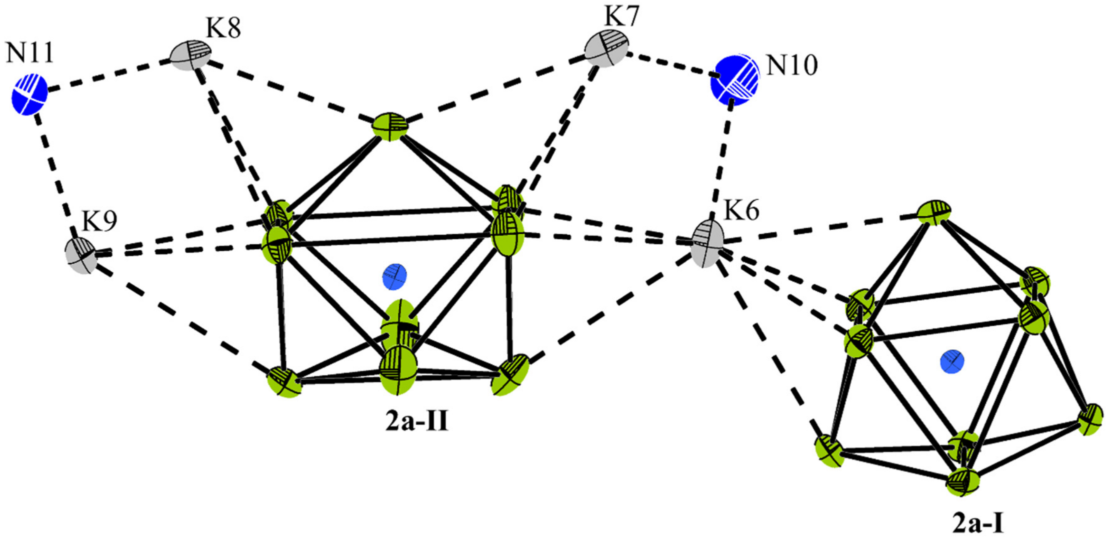

Figure S5. Partial coordination spheres of $[Ru@Sn_9]^{6-}$ in **2** with neighboring potassium atoms and ammonia molecules; H atoms are omitted for clarity; displacement ellipsoids are displayed with a 50 % probability.

Table S2. Interatomic *E-E* distances in Co-centered and in empty Ge_9 clusters in the title compound (**1a**) and in the related compound $K_5[OH][Ge_9] \cdot 11\ NH_3$[3] (**3**) as well as in Co-centered and in empty $[Sn_9]$ clusters in $[K(2.2.2)crypt]_3KCo_{0.68}Sn_9$[4] and in $[K(2.2.2)crypt]_3KSn_9$[5], respectively; for both pairs an elongation factor of *E-E* distances of filled clusters compared to the respective empty clusters is given. The atom labelling is chosen in a way that all Ge_9 as well as Sn_9 clusters show the same orientation.

	**1a**	**3**			$[Co_{0.68}@Sn_9]^{4-}$	$[Sn_9]^{4-}$	
Atoms	Distances	Distances	Ratio	Atoms	Distances	Distances	Ratio
Ge1-Ge2	2.6826(9)	2.5965	1.033	Sn1-Sn2	3.0342(4)	2.931	1.035
Ge2-Ge3	2.7283(9)	2.5863	1.055	Sn2-Sn3	3.0365(4)	2.949	1.030
Ge3-Ge2^i	2.7283(9)	2.5863	1.055	Sn3-Sn4	3.0736(4)	2.969	1.035
Ge2^i-Ge1	2.6826(9)	2.5965	1.033	Sn4-Sn1	3.1008(4)	2.973	1.043
Ge1-Ge3	3.5719(13)	3.6071	0.990	Sn1-Sn3	4.3595(5)	4.230	1.031
Ge2-Ge2^i	4.0115(13)	3.7204	1.078	Sn2-Sn4	4.2976(4)	4.129	1.041
Ge1-Ge5	2.7474(10)	2.5780	1.065	Sn1-Sn5	2.9817(4)	2.950	1.011
Ge2-Ge5	2.7296(10)	2.5892	1.054	Sn2-Sn5	2.9851(4)	2.9493	1.008
Ge2-Ge4	2.7203(11)	2.5824	1.053	Sn2-Sn6	3.0173(4)	2.9644	1.014
Ge3-Ge4	2.6918(11)	2.5949	1.037	Sn3-Sn6	3.0339(4)	2.9777	1.017
Ge3-Ge4^i	2.6918(11)	2.5949	1.037	Sn3-Sn7	2.9990(4)	2.9549	1.013
Ge2-Ge4^i	2.7203(11)	2.5824	1.053	Sn4-Sn7	3.0582(4)	2.9957	1.027
Ge2-Ge5^i	2.7296(10)	2.5892	1.054	Sn4-Sn8	2.9983(4)	2.9527	1.012
Ge1-Ge5^i	2.7474(10)	2.5780	1.065	Sn1-Sn8	3.0436(4)	2.9850	1.016
Ge5-Ge4	3.4832(10)	2.8501	1.222	Sn5-Sn6	3.2506(4)	3.1249	1.025
Ge4-Ge4^i	2.8105(14)	2.7907	1.007	Sn6-Sn7	3.3260(4)	3.2267	1.038
Ge4^i-Ge5^i	3.4832(10)	2.8501	1.222	Sn7-Sn8	3.3330(4)	3.1714	1.036
Ge5-Ge5^i	2.7853(15)	2.7811	1.002	Sn8-Sn5	3.4350(4)	3.3110	1.040
Ge5^i-Ge6	2.7362(11)	2.5794	1.061	Sn5-Sn9	2.9712(4)	2.9453	1.005
Ge4^i-Ge6	2.6946(10)	2.5790	1.045	Sn6-Sn9	2.9479(4)	2.9283	1.004
Ge4-Ge6	2.6946(10)	2.5790	1.045	Sn7-Sn9	2.9911(4)	2.9613	1.006
Ge5-Ge6	2.7362(11)	2.5794	1.061	Sn8-Sn9	2.9636(4)	2.9327	1.006
Co-Ge1	2.3589(14)	-		Co-Sn1	2.5601(8)	-	
Co-Ge2	2.3469(8)	-		Co-Sn2	2.5745(7)	-	
Co-Ge3	2.3630(13)	-		Co-Sn3	2.5717(8)	-	
Co-Ge2^i	2.3469(8)	-		Co-Sn4	2.5533(7)	-	
Co-Ge4	2.3638(11)	-		Co-Sn5	2.6065(7)	-	
Co-Ge5	2.3735(10)	-		Co-Sn6	2.6015(7)	-	
Co-Ge5^i	2.3735(10)	-		Co-Sn7	2.5959(7)	-	
Co-Ge4^i	2.3638(11)	-		Co-Sn8	2.5848(7)	-	
Co-Ge6	2.3311(14)	-		Co-Sn9	2.8859(7)	-	

Symmetry operations: (i) for compound **1**: *x*, 0.5-*y*, *z*; (i) for compound **3**: *x*, -*y*, *z*.

Table S3. Interatomic distances in Ru-centered and in empty [Sn_9] clusters in the title compound (**2**) and in the related compound $K_5[OH][Sn_9] \cdot 11\ NH_3$ (**4**) , respectively.[6]

**2a-I**		**2a-II**		**4**	
Atoms	Distance / Å	Atoms	Distance / Å	Atoms	Distance / Å
Sn3-Sn4	3.0926(6)	Sn11-$Sn9^{i}$	3.1095(9)	Sn1-Sn2	2.9557(4)
Sn4-$Sn3^{i}$	3.0925(6)	$Sn9^{i}$-Sn7	3.0525(9)	Sn2-Sn3	2.9440(4)
$Sn3^{i}$-Sn6	3.1157(6)	Sn7-Sn9	3.0525(9)	Sn3-$Sn2^{ii}$	2.9440(4)
Sn6-Sn3	3.1157(6)	Sn9-Sn11	3.1095(9)	$Sn2^{ii}$-Sn1	2.9557(4)
Sn4-Sn6	4.5728(8)	Sn9-$Sn9^{i}$	4.6219(14)	Sn2-$Sn2^{ii}$	4.2247(5)
Sn3-$Sn3^{i}$	4.1157(8)	Sn7-Sn11	3.9434(12)	Sn1-Sn3	4.1164(6)
Sn6-Sn2	3.0909(6)	$Sn9^{i}$-$Sn12^{i}$	3.0544(8)	Sn2-Sn4	2.9515(5)
Sn6-$Sn2^{i}$	3.0909(6)	$Sn9^{i}$-$Sn8^{i}$	3.0598(9)	Sn2-Sn5	2.9542(4)
Sn4-Sn1	3.0934(6)	Sn7-$Sn8^{i}$	3.1300(10)	$Sn2^{ii}$-$Sn4^{ii}$	2.9515(5)
Sn4-$Sn1^{i}$	3.0934(6)	Sn7-Sn8	3.1300(10)	$Sn2^{ii}$-$Sn5^{ii}$	2.9542(4)
Sn3-Sn2	3.0761(6)	Sn9-Sn8	3.0598(9)	Sn1-Sn5	2.9538(4)
Sn3-Sn1	3.0679(6)	Sn9-Sn12	3.0544(8)	Sn1-$Sn5^{ii}$	2.9538(4)
$Sn3^{i}$-$Sn2^{i}$	3.0761(6)	Sn11-Sn12	3.0767(8)	Sn3-Sn4	2.9776(4)
$Sn3^{i}$-$Sn1^{i}$	3.0679(6)	Sn11-$Sn12^{i}$	3.0768(8)	Sn3-$Sn4^{ii}$	2.9776(4)
Sn1-Sn2	3.1409(6)	Sn12-$Sn12^{i}$	3.1192(9)	Sn5-$Sn5^{ii}$	3.1582(4)
Sn2-$Sn2^{i}$	4.0697(9)	$Sn12^{i}$-$Sn8^{i}$	4.0207(7)	$Sn5^{ii}$-$Sn4^{ii}$	3.2245(4)
$Sn2^{i}$-$Sn1^{i}$	3.1409(6)	$Sn8^{i}$-Sn8	3.0874(10)	$Sn4^{ii}$-Sn4	3.1363(4)
$Sn1^{i}$-Sn1	4.0319(9)	Sn8-Sn12	4.0207(7)	Sn4-Sn5	3.2245(4)
Sn2-Sn5	3.0845(6)	Sn8-Sn10	3.0512(8)	Sn4-Sn6	2.9353(5)
Sn1-Sn5	3.0888(6)	$Sn8^{i}$-Sn10	3.0512(8)	$Sn4^{ii}$ -Sn6	2.9353(4)
$Sn2^{i}$-Sn5	3.0845(6)	Sn12-Sn10	3.1115(7)	Sn5-Sn6	2.9346(4)
$Sn1^{i}$-Sn5	3.0887(6)	$Sn12^{i}$-Sn10	3.1115(7)	$Sn5^{ii}$ -Sn6	2.9346(4)
Ru1-Sn1	2.7101(6)	Ru2-Sn8	2.6987(7)	-	-
Ru1-$Sn1^{i}$	2.7101(6)	Ru2-$Sn8^{i}$	2.6987(7)	-	-
Ru1-Sn2	2.7173(6)	Ru2-Sn11	2.6711(9)	-	-
Ru1-$Sn2^{i}$	2.7173(6)	Ru2-Sn12	2.6867(6)	-	-
Ru1-Sn3	2.7001(5)	Ru2-$Sn12^{i}$	2.6867(6)	-	-
Ru1-$Sn3^{i}$	2.7001(5)	Ru2-Sn10	2.6406(9)	-	-
Ru1-Sn4	2.6528(8)	Ru2-Sn7	2.6577(9)	-	-
Ru1-Sn5	2.6120(8)	Ru2-Sn9	2.6414(6)	-	-
Ru1-Sn6	2.6394(8)	Ru2-$Sn9^{i}$	2.6414(6)	-	-

Symmetry operations: (i): *x*, 0.5-*y*, *z*; (ii): *x*, -*y*, *z*.

Table S4. Comparison of the volume increase across empty/filled $[E_9]^{n-}$ clusters (E = Ge, Sn, Pb; n = 3; 4; 5; 6).

Cluster	Volume / Å^3	Volume increase compared to empty cluster	Approx. Symmetry	Reference
$[Ge_9]^{4-}$	22.1		C_{4v}	[3]
$[Ni@Ge_9]^{3-}$ [a]	25.4	15.1 %	C_s	[7]
$[Co@Ge_9]^{5-}$ - **1a**	26.4	19.4 %	D_{3h}	this work
$[Sn_9]^{4-}$	32.9		C_{4v}	[8]
$[Ru@Sn_9]^{6-}$ **- 2a**	38.3	16.4 %	D_{3h}	this work
$[Ru@Sn_9]^{6-}$ **- 2b**	38.8	17.9 %	D_{3h}	this work
$[Co_{0.68}@Sn_9]^{4.68-}$	35.4	7.7 %	C_{4v}	[9]
$[Co_{0.79}@Sn_9]^{4.79-}$	35.3	7.6 %	C_{4v}	[4]
$[Ni@Sn_9]^{4-}$	34.8	5.9 %	C_{4v}	[10]
$[Cu@Sn_9]^{3-}$	36.4	10.6 %	C_{4v}	[11]
$[Cu@Sn_9]^{3-}$	37.3	13.4 %	D_{3h}	[12]
$[Pb_9]^{4-}$	37.4		C_{4v}	[8]
$[Cu@Pb_9]^{3-}$	41.7	11.3%	D_{3h}	[12]

[a] Mean value of two crystallographically independent cluster units.

Table S5. Range of interatomic distances between transition metal and tetrel atoms in$[Co@Ge_9]^{5-}$ and $[Ru@Sn_9]^{6-}$ and of other selected endohedral clusters.

	d(*TM*-*E*) / Å		d(*TM*-*E*) / Å
$[Co@Ge_9]^{5-}$ - **1a**	2.331(1) - 2.374(1)	$[Ru@Sn_9]^{6-}$ **- 2a**	2.612(2) - 2.711(1)
$[Ni@Ge_9]^{4-}$ [7]	2.267(3) - 2.410(2)	$[Ru@Ge_{12}]^{3-}$ [13]	2.651(1) - 2.771(1)
$[Co@Ge_{10}]^{3-}$ [14]	2.479(1) - 2.525(1)	$[Co_{0.68}@Sn_9]^{4-}$ [9]	2.553(1) - 2.886(1)
$[Co_{0.68}@Sn_9]^{4-}$ [9]	2.5533(7) - 2.8859(7)	$[Co_{0.79}@Sn_9]^{4.79-}$ [4]	2.518(2) - 2.702(2)
$[Co_{0.79}@Sn_9]^{4.79-}$ [4]	2.518(2) - 2.702(2)	$[Ni@Sn_9]^{4-}$ [10]	2.487(3) - 2.784(2)
		$[Cu@Sn_9]^{3-}$ [12]	2.611(7) - 2.700(7)

3. Computational Analysis

The computational analysis for $[Co@Ge_9]^{5-}$, $[Ru@Sn_9]^{6-}$, $[Ge_9]^{4-}$, and $[Sn_9]^{4-}$ were performed using the Gaussian09 program package,[15] with exchange correlation hybrid functional after Perdew, Burke and Ernzerhof (PBE0)[16] and def2-TZVPP basis sets for the considered elements Ge,[17] Co,[18] Ru,[19] and Sn.[19b, 20] For compensation of the negative charge, a solvation model (polarizable continuum model, PCM)[18] based on water was estimated for all calculations. The structures were optimized, and the characters of the stationary points were investigated by frequency calculations. $[Ru@Sn_9]^{6-}$ reveals a minor imaginary frequency (17.8i cm^{-1}). The imaginary frequency of the highly charged clusters probably arise from the fact that the missing crystal surroundings are replaced by a solvation model. Hirshfeld and natural charges were calculated for $[Co@Ge_9]^{5-}$and $[Ru@Sn_9]^{6-}$.[21] For a molecular orbital diagram single point calculations of $[Ge_9]^{4-}$ and $[Sn_9]^{4-}$ in D_{3h} symmetry were carried out. For Co^- and Ru^{2-} idealized orbitals were taken to construct the MO-scheme.

Additionally, Raman intensities were calculated for all filled and empty clusters (see Tables S6 and S8). For data processing and visualization Jmol,[22] VESTA 3[2], IBOview[23] and Origin 9.1[24] were used. Calculated Raman shifts are subject to change using appropriate scale factors.[25]

Table S6. Hirshfeld, natural, and Mulliken charges of $[Co@Ge_9]^{5-}$ and $[Ru@Sn_9]^{6-}$.

	$Co@Ge_9]^{5-}$			$[Ru@Sn_9]^{6-}$		
	Hirshfeld	Natural charge	Mulliken*	Hirshfeld	Natural charge	Mulliken*
Average of Sn/Ge atoms	-0.50	-0.16	-0.67	-0.64	-0.3	-0,47
Co/Ru	-0.53	-3.56	1.07	-0.25	-3.32	-1.73

* In general, Mulliken charges are highly dependent on the basis set and may lead to some population inversions.

Table S7. Comparison of observed and optimized parameters of $[Ge_9]^{4-}$ and $[Co@Ge_9]^{5-}$. The structures with D_{3h} symmetry are taken from the optimized structures of the filled clusters.

	$[Ge_9]^{4-}$ [3] (measured)	$[Ge_9]^{4-}$ (optimized)	$[Ge_9]^{4-}$ (single point)	$[Co@Ge_9]^{5-}$ (measured)	$[Co@Ge_9]^{5-}$ (optimized)
Point group	C_{4v}	C_{4v}	D_{3h}	D_{3h}	D_{3h}
band gap / eV	/	4.35	3.66	/	3.73
d(Ge-Ge) / Å	2.5780(6) - 2.8501(6)	2.58 - 2.83	2.71 - 2.76	2.6826(9) - 2.8105(14)	2.71 - 2.76
d(Co-Ge) / Å	/	/	/	2.3311(14) - 2.3735(10)	2.35 - 2.36

Table S8. Comparison of observed and optimized parameters of $[Sn_9]^{4-}$ and $[Ru@Sn_9]^{6-}$. The structures with D_{3h} symmetry are taken from the optimized structures of the filled clusters.

	$[Sn_9]^{4-}$ [3] (measured)	$[Sn_9]^{4-}$ (optimized)	$[Sn_9]^{4-}$ (single point)	$[Ru@Sn_9]^{6-}$ (measured)	$[Ru@Sn_9]^{6-}$ (optimized)
point group	C_{4v}	C_{4v}	D_{3h}	D_{3h}	D_{3h}
band gap / eV	/	3.53	2.98	/	2.89
d(Sn-Sn) / Å	2.9346(4) - 3.2245(4)	2.96 - 3.22	3.09 - 3.14	3.0512(8) - 3.1409(6)	3.09 - 3.14
d(Ru-Sn) / Å	/	/	/	2.6120(8) - 2.7173(6)	2.66 - 2.69

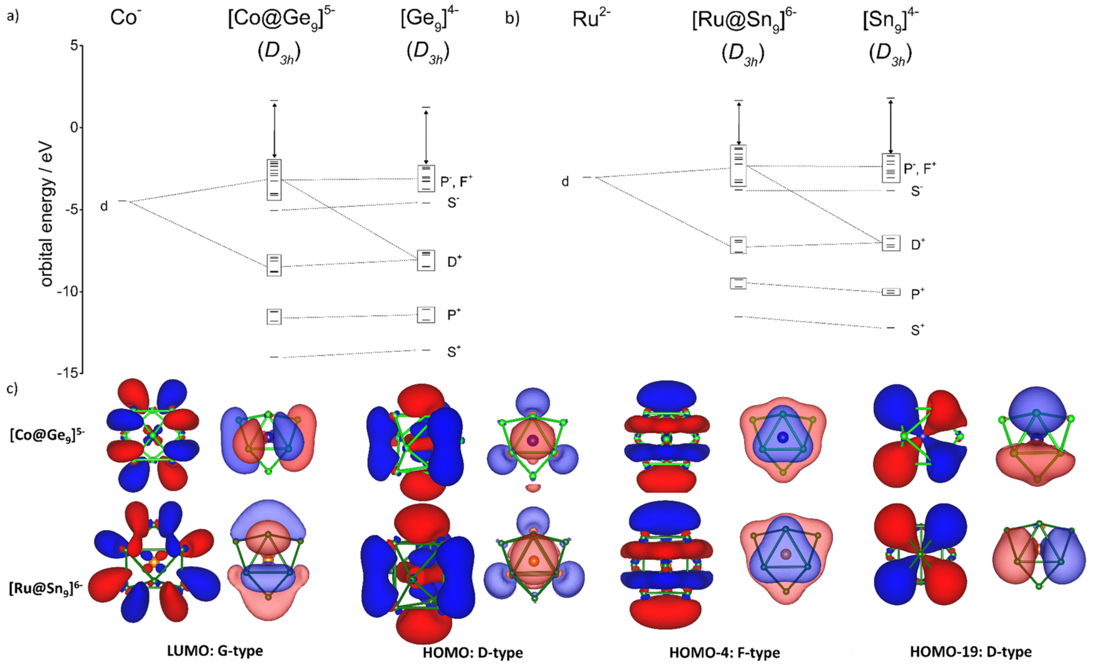

Figure S6. Molecular orbital interaction diagram of $[Co@Ge_9]^{5-}$ (a) in fragments of Co^- and $[Ge_9]^{4-}$ (D_{3h} symmetry) and of $[Ru@Sn_9]^{6-}$ (b) in fragments of Ru^{2-} and $[Sn_9]^{4-}$ (D_{3h} symmetry). Important contributions are indicated by dashed lines of both molecular orbital interaction diagrams. c) Relevant molecular orbitals of $[Co@Ge_9]^{5-}$ and $[Ru@Sn_9]^{6-}$ with iso-values 0.025 and 0.02, respectively. Each orbital shows a specific shape of D, F, and G orbital type.

Table S9. Atom contribution for IBOs per 2 electrons and in percentage for $[Co@Ge_9]^{5-}$.

	Co6	Ge1	Ge2	Ge3	Ge4	Ge5	Ge7	Ge8	Ge9	Ge10	other
IBO1	1.732	0	0.024	0.063	0	0.116	0	0.043	0	0	0.022
IBO2	1.736	0	0.056	0	0.117	0	0	0.048	0	0	0.043
IBO3	1.745	0	0.032	0	0	0	0.111	0.021	0	0.065	0.026
IBO4	1.561	0.228	0	0.047	0	0	0	0.06	0	0.065	0.038
IBO5	1.559	0	0.058	0.056	0	0	0	0	0.218	0.082	0.027
Overall	8.333	0.228	0.17	0.166	0.117	0.116	0.111	0.172	0.218	0.212	0.156
Overall [%]	83.33	2.28	1.7	1.66	1.17	1.16	1.11	1.72	2.18	2.12	1.56

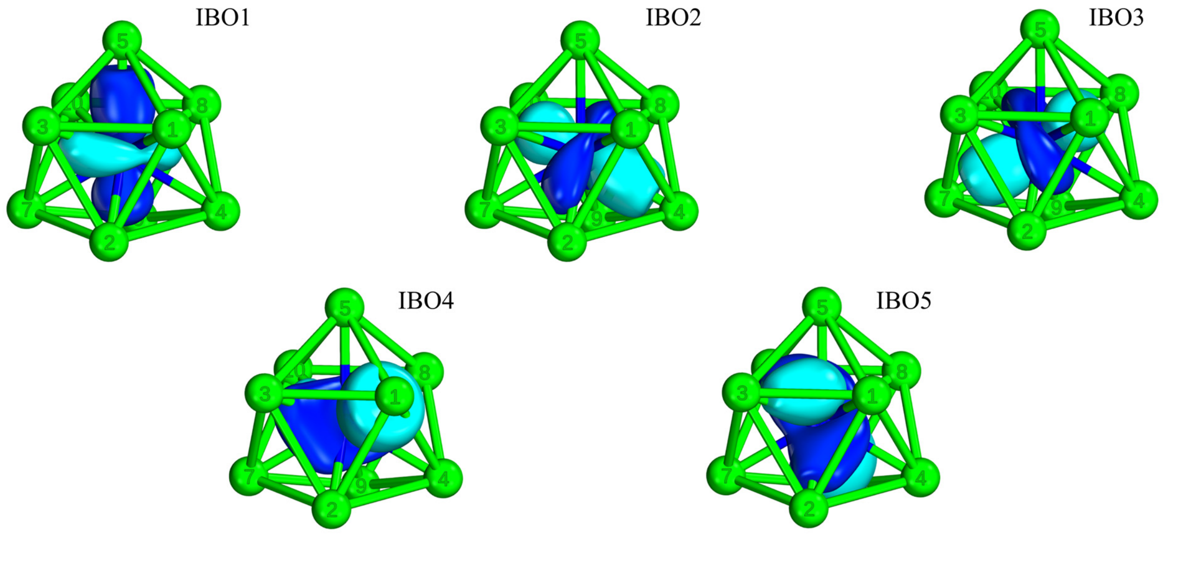

Figure S7. IBOs concerning the central atom of $[Co@Ge_9]^{5-}$. Co and Ge is shown in blue and light green, respectively.

Table S10. Atom contribution for IBOs per 2 electrons and in percentage for $[Ru@Sn_9]^{6-}$.

	Ru10	Sn1	Sn2	Sn3	Sn4	Sn5	Sn6	Sn7	Sn8	Sn9	other
IBO1	1.605	0.175	0	0	0	0	0.105	0	0	0.087	0.027
IBO2	1.583	0	0.149	0	0	0	0.028	0.173	0	0.052	0
IBO3	1.569	0	0	0	0.198	0.136	0.044	0	0	0.035	0
IBO4	1.492	0	0	0.371	0.03	0	0	0.031	0	0.02	0.055
IBO5	1.485	0	0	0	0.026	0	0	0.031	0.386		0.072
Overall	7.734	0.175	0.149	0.371	0.254	0.136	0.177	0.235	0.386	0.194	0.154
Overall [%]	77.34	1.75	1.49	3.71	2.54	1.36	1.77	2.35	3.86	1.94	1.54

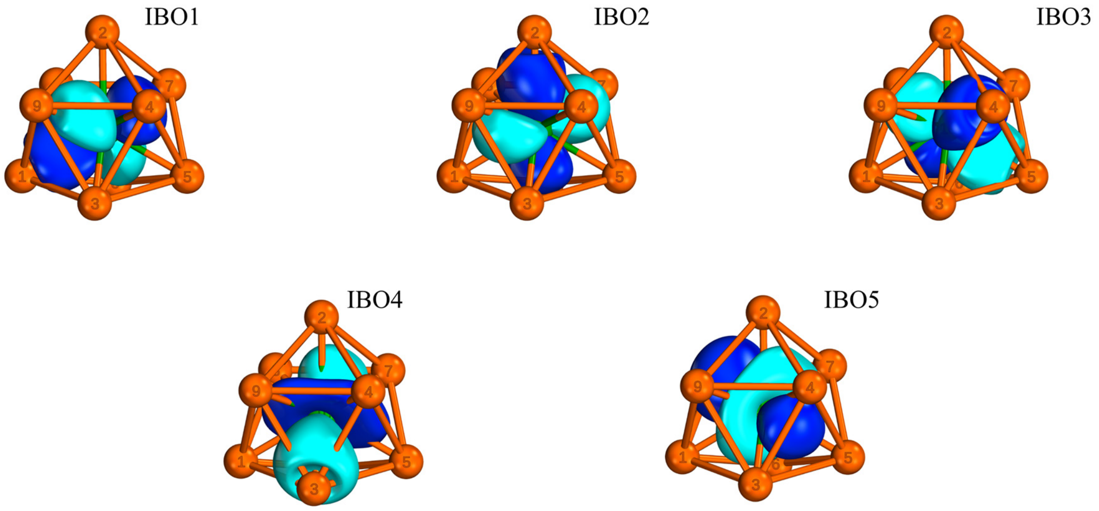

Figure S8. IBOs concerning the central atom of $[Ru@Sn_9]^{6-}$. Ru and Sn is shown in green and orange, respectively.

Table S11. Raman modes of the $[Ge_9]^{4-}$ (meas. and calc.) and $[Co@Ge_9]^{5-}$ clusters.

	$[Ge_9]^{4-}$ [26]	$[Ge_9]^{4-}$ (calc.)	$[Co@Ge_9]^{5-}$ (calc.)
			47.94
		10.44	68.36
		84.23	72.55
		84.51	104.89
		132.22	109.45
		133.48	110.47
		142.85	120.57
		149.75	123.98
	104	149.92	124.14
	125	153.79	127.38
Raman shift / cm^{-1}	147	157.98	129.20
	164	161.69	158.96
	188	161.81	159.39
	220	177.24	167.69
	241	194.09	199.00
		194.25	200.86
		197.79	203.43
		217.98	207.06
		225.35	207.64
		228.44	244.90
		228.49	358.48
		241.49	361.93
			398.39

Table S12. Raman modes of the $[Sn_9]^{4-}$ (meas. and calc.) and $[Ru@Sn_9]^{6-}$ clusters.

	$[Sn_9]^{4-}$ [26]	$[Sn_9]^{4-}$ (calc.)	$[Ru@Sn_9]^{6-}$ (calc.)
Raman shift / cm^{-1}	99<br>146	9.5752<br>56.0156<br>56.0578<br>84.4421<br>89.1948<br>92.1377<br>94.0746<br>99.5294<br>99.5879<br>105.8489<br>108.4646<br>108.4839<br>118.897<br>130.3429<br>130.5298<br>130.5616<br>145.6974<br>148.6258<br>153.71<br>153.7661<br>164.0781	4.81935<br>50.7005<br>58.5891<br>60.97031<br>79.88666<br>81.50827<br>83.15437<br>85.5199<br>86.98353<br>87.39189<br>89.50889<br>104.88136<br>111.32339<br>119.99434<br>136.54036<br>136.9353<br>138.12973<br>141.2574<br>144.3542<br>171.07194<br>260.3126<br>261.61051<br>280.682

4. X-Ray powder diffraction (PXRD)

Data were collected at room temperature on a STOE Stadi P diffractometer (Cu $K_{\alpha 1}$ radiation, Ge(111) monochromator) with a Dectris MYTHEN 1K detector in Debye-Scherrer geometry. Samples were sealed in glass capillaries (Ø 0.3 mm) for measurement. Raw data were processed with WinXPOW.[27]

The analyses show characteristic reflections in the 2θ range of 10° to 20° indicating the presence of clusters in the precursor phases (Figure S9). The best accordance of the synthesized phases is obtained with the corresponding $K_{12}E_{17}$ phases (E = Ge, Sn). PXRD analyses prove the absence of clathrates or elemental tetrels.

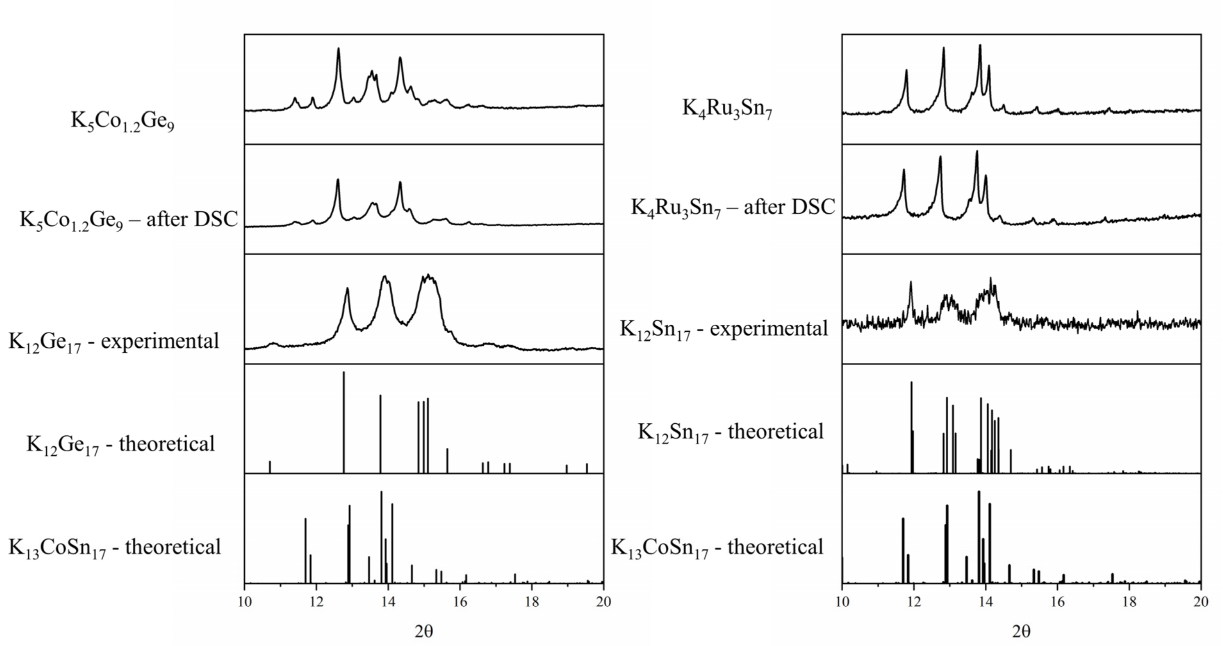

Figure S9. X-ray powder patterns (Cu $K_{\alpha 1}$) of the precursor phase of **1** ("$K_5Co_{1.2}Ge_9$") and of **2** ("$K_4Ru_3Sn_7$"). Both precursors show the best accordance with $K_{12}E_{17}$ phases of the respective tetrel elements as well as to $K_{13}CoSn_{17}$ (for the Ge phase with smaller lattice parameters). Both phases show only slight changes after DSC analyses up to 1000 °C.

5. Raman spectroscopy

Raman spectra between 50 and 800 cm^{-1} of crystalline samples sealed in glass capillaries were recorded at ambient temperature using a Renishaw inVia Raman Microscope RE04 (August 2015) equipped with a CCD Detector and WiRE 4.2 build 5037, Renishaw 2002 software. The samples were either measured using a 785 nm or a 532 nm laser.

In the Raman spectrum of the precursor of **1**, "$K_5Co_{1.2}Ge_9$", several strong modes appear in a range which matches the strongest calculated modes of the $[Co@Ge_9]^{5-}$ cluster (207 cm^{-1}, 244 cm^{-1}) and of K_4Ge_9 (143, 165, 185 and 222 cm^{-1}). Therefore we assume the presence of both, the endohedral species and the empty $[Ge_9]^{4-}$ cluster, in the precursor. Unfortunately, some binary K-Ge phases,[26] clathrates[28] and elemental Ge modifications[29] also exhibit signals in this range. However, none of the mentioned phases show signals in the region around 360 cm^{-1}, where a relatively weak mode is detected in the spectrum of **1**. This matches to the calculated modes of $[Co@Ge_9]^{5-}$ which originate from two vibrations of the endohedral Co atom and several Ge atoms, the "A" and "B" modes in Figure S6, and, thus, are a direct result of the endohedral nature of the cluster. Therefore, the mode at 360 cm^{-1} serves as an unequivocal hint for the presence of the endohedral cluster in the precursor.

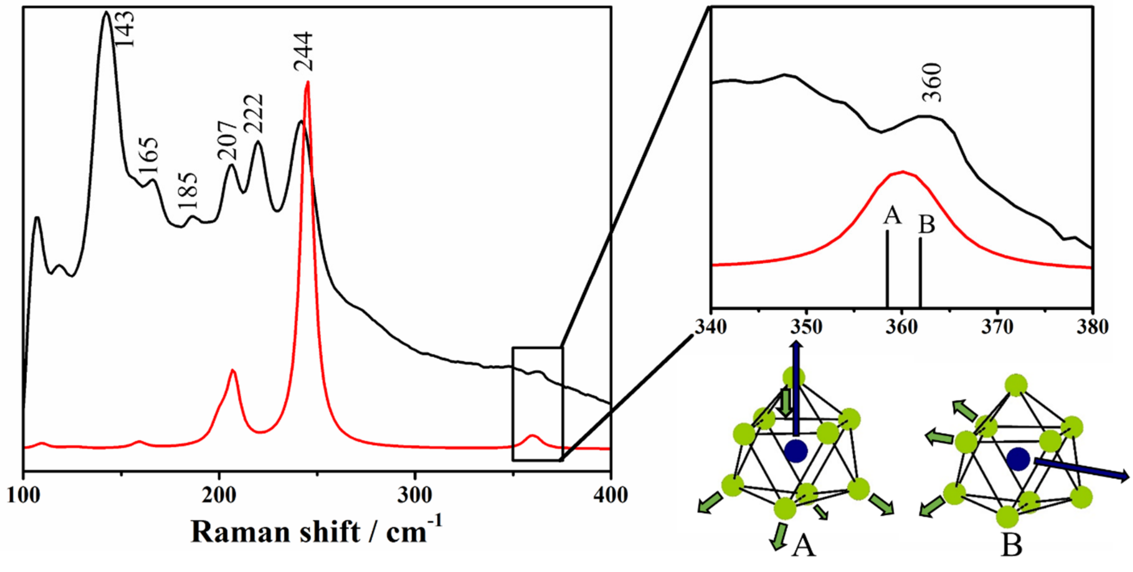

Figure S10. Raman spectrum of "$K_5Co_{1.2}Ge_9$" (black) and the calculated spectrum of **1a** (red) with characteristic endohedral modes (**A**, **B**) at 360 cm^{-1}.

The spectrum of the precursor of **2**, "$K_4Ru_3Sn_7$", reflects a similar, but less clear situation. While the main $[Sn_9]^{4-}$ mode is not present, the strongest calculated mode for the $[Ru@Sn_9]^{6-}$ cluster is shifted to smaller wavenumbers by about 10 cm^{-1} from the largest signal of the spectrum. However, the corresponding "A" and "B" vibrations for $[Ru@Sn_9]^{6-}$ appear at 260 cm^{-1} in a region free of possible modes of any related binary compounds,[26, 30] clathrates[28c, 31] or elemental tin modifications,[32] and hint for the presence of the endohedral cluster $[Ru@Sn_9]^{6-}$ in the solid precursor.

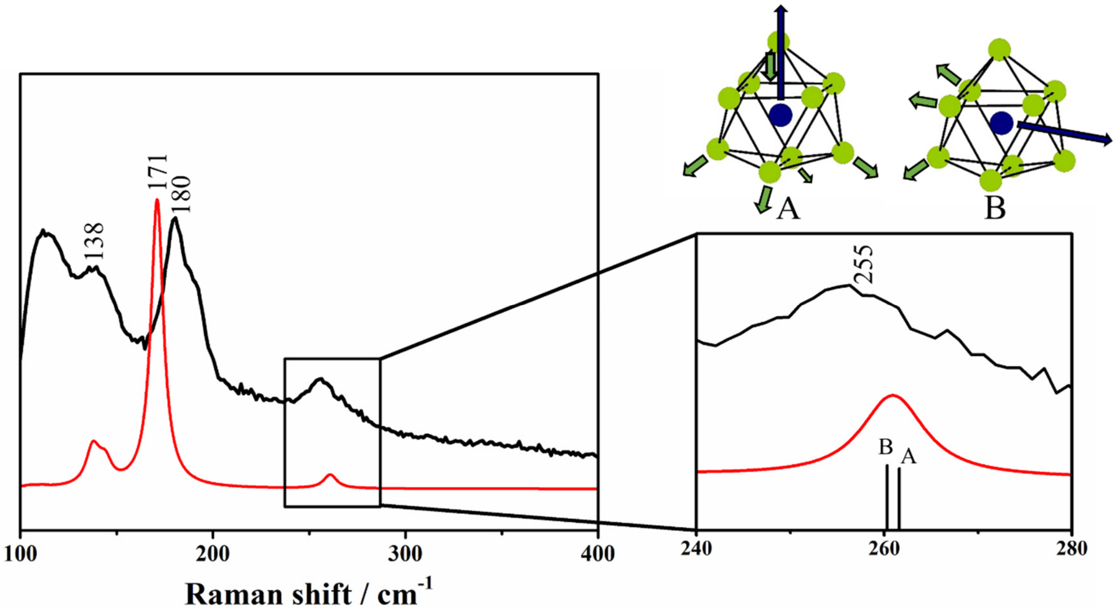

Figure S11. Raman spectrum of "$K_4Ru_3Sn_7$" (black) and calculated spectrum of **2a** (red) with characteristic endohedral modes (**A**, **B**) at 255 cm^{-1}.

Raman spectra of precipitates which are obtained after evaporation of NH_3 from solutions of the precursors of **1** and **2** show no differences compared to the spectra recorded before dissolution.

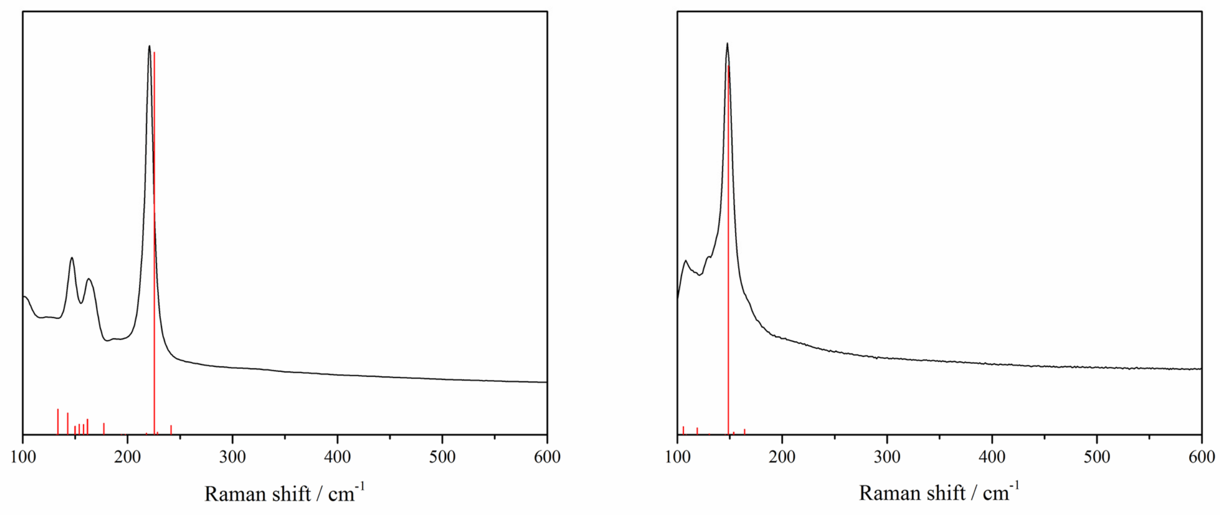

Figure S12. Raman spectra of empty [E_9] (E = Ge, Sn) clusters. Black lines – experimental spectra of K_4Ge_9 (left) and K_4Sn_9 (right). Red lines – Raman shifts and intensities calculated using DFT.

6. Differential Scanning Calorimetry (DSC)

DSC analyses between room temperature and 1000 °C were recorded in sealed Nb-ampules on a Netzsch DSC 404 Pegasus device. Empty sealed crucibles served as a reference. Measurements were performed under an Ar flow of 60 - 70 mL/min and a heating/cooling rate of 10 °C/min. Data collection and handling was carried out with the Proteus Thermal Analysis program.

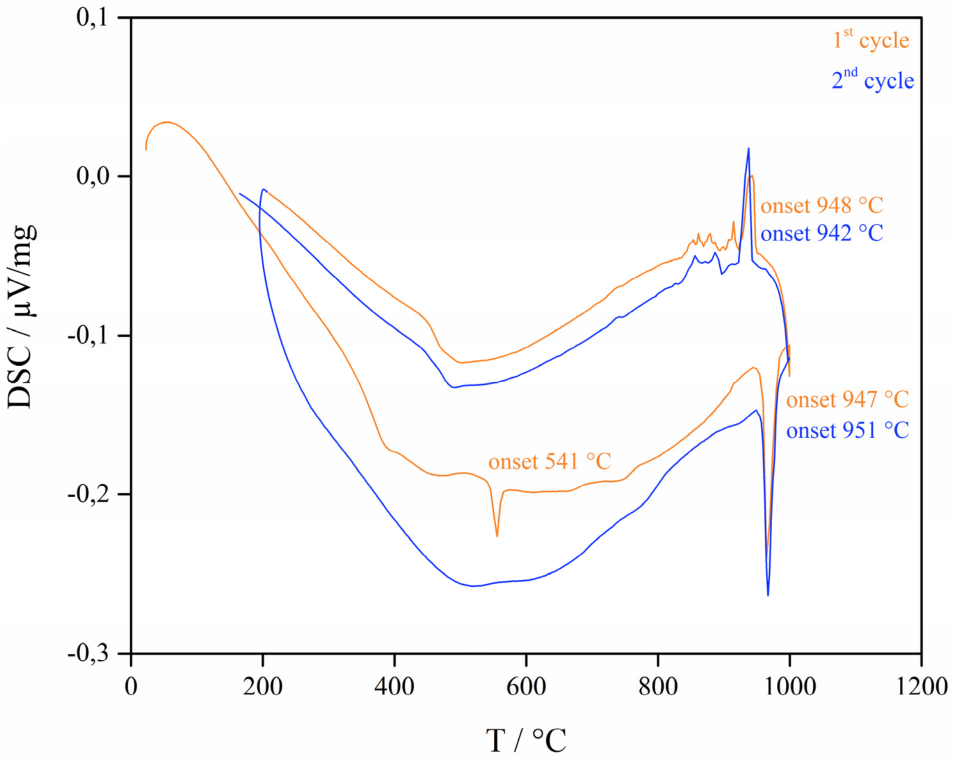

Figure S13. DSC analysis of "$K_5Co_{1.2}Ge_9$"between room temperature and 1000 °C.

The DSC analysis of "$K_5Co_{1.2}Ge_9$" shows three distinct signals (Figure S12). The onset temperature of 950 °C of two reversible signals coincides with the melting and recrystallization of CoGe.[33] One signal with an onset of 541 °C is only seen during the first heating cycle and can be assigned to the degradation of the $[Co@Ge_9]^{5-}$ clusters which, according to Raman spectroscopy, cannot be detected after the heating cycles. After heating of the sample to 1000 °C, the modes of the filled cluster disappeared, while the main modes of $[Ge_9]^{4-}$ are still visible (Figure S13). A DSC analysis of "$K_4Ru_3Sn_7$" shows no signals, and Raman spectra of this compound are almost identical before and after heating (Fig. S14).

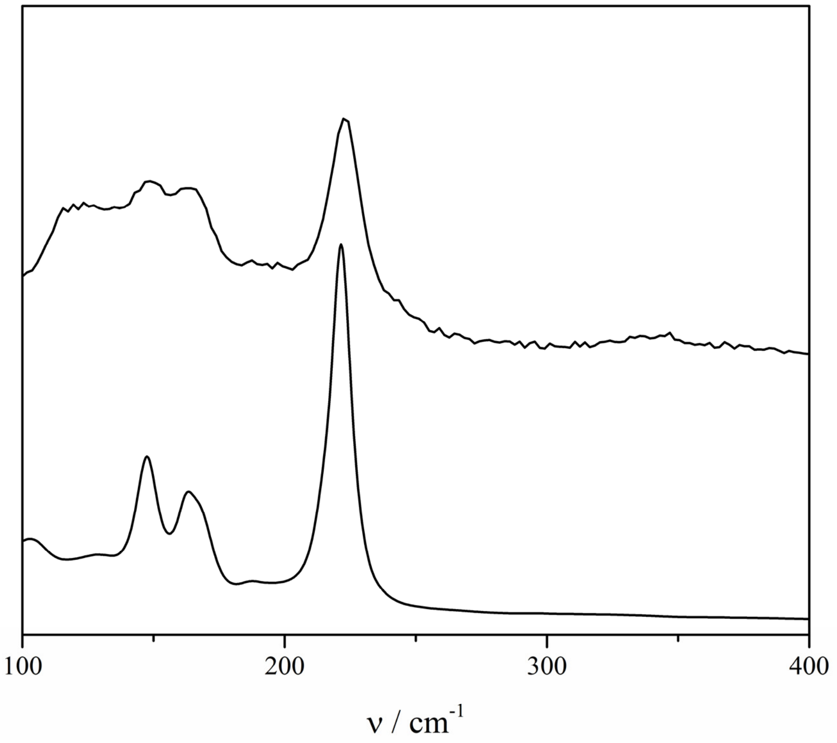

Figure S14. Raman spectra of "$K_5Co_{1.2}Ge_9$" after DSC to 1000 °C (above) and of K_4Ge_9 as a reference (below).

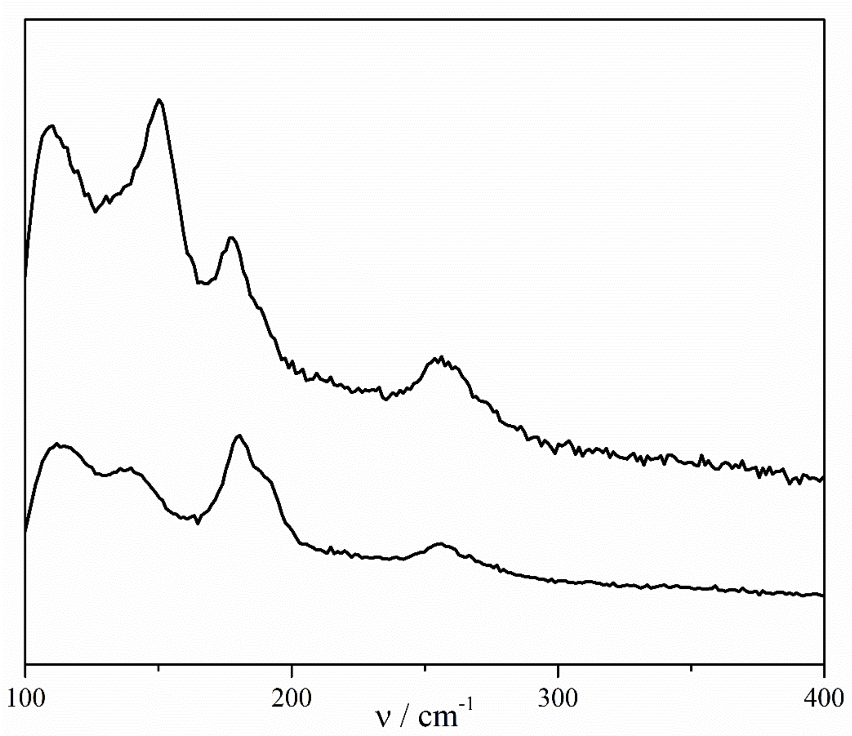

Figure S15. Raman spectra of "$K_4Ru_3Sn_7$" after DSC to 1000 °C (above) and before (below).

7. References

[1] G. M. Sheldrick, *Acta Cryst.* **2008**, *64*, 112.
[2] K. Momma, F. Izumi, *J. Appl. Crystallogr.* **2011**, *44*, 1272.
[3] C. B. Benda, H. He, W. Klein, M. Somer, T. F. Fässler, *Z. Anorg. Allg. Chem.* **2015**, *641*, 1080.
[4] V. Hlukhyy, H. He, L.-A. Jantke, T. F. Fässler, *Chem. Eur. J.* **2012**, *18*, 12000.
[5] T. F. Fässler, R. Hoffmann, *CHIMIA* **1998**, *52*, 158.
[6] K. Mayer, J. V. Dums, W. Klein, T. F. Fässler, *Angew. Chem. Int. Ed.* **2017**, *56*, 15159; *Angew. Chem.* **2017**, *129*, 15356.
[7] J. M. Goicoechea, S. C. Sevov, *Angew. Chem. Int. Ed.* **2005**, *44*, 4026; *Angew. Chem.* **2005**, *117*, 4094.
[8] N. Korber, A. Fleischmann, *J. Chem. Soc., Dalton Trans.* **2001**, 383.
[9] H. He, W. Klein, L.-A. Jantke, T. F. Fässler, *Z. Anorg. Allg. Chem.* **2014**, *640*, 2864.
[10] M. M. Gillett-Kunnath, J. I. Paik, S. M. Jensen, J. D. Taylor, S. C. Sevov, *Inorg. Chem.* **2011**, *50*, 11695.
[11] S. Scharfe, Dissertation, Technical University of Munich (Munich), **2010**.
[12] S. Scharfe, T. F. Fässler, S. Stegmaier, S. D. Hoffmann, K. Ruhland, *Chem. Eur. J.* **2008**, *14*, 4479.
[13] G. Espinoza-Quintero, J. C. A. Duckworth, W. K. Myers, J. E. McGrady, J. M. Goicoechea, *J. Am. Chem. Soc.* **2014**, *136*, 1210.
[14] J.-Q. Wang, S. Stegmaier, T. F. Fässler, *Angew. Chem. Int. Ed.* **2009**, *48*, 1998; *Angew. Chem.* **2009**, *121*, 2032.
[15] Gaussian09, M. J. Frisch, G. W. Trucks, H. B. Schlegel, G. E. Scuseria, J. R. C. M. A. Robb, G. Scalmani, V. Barone, B. Mennucci, G. A. Petersson, H. Nakatsuji, M. Caricato, X. Li, H. P. Hratchian, A. F. Izmaylov, J. Bloino, G. Zheng, J. L. Sonnenberg, M. Hada, M. Ehara, K. Toyota, R. Fukuda, J. Hasegawa, M. Ishida, T. Nakajima, Y. Honda, O. Kitao, H. Nakai, T. Vreven, J. A. Montgomery Jr., J. E. Peralta, F. Ogliaro, M. Bearpark, J. J. Heyd, E. Brothers, K. N. Kudin, V. N. Staroverov, T. Keith, R. Kobayashi, J. Normand, K. Raghavachari, A. Rendell, J. C. Burant, S. S. Iyengar, J. Tomasi, M. Cossi, N. Rega, J. M. Millam, M. Klene, J. E. Knox, J. B. Cross, V. Bakken, C. Adamo, J. Jaramillo, R. Gomperts, R. E. Stratmann, O. Yazyev, A. J. Austin, R. Cammi, C. Pomelli, J. W. Ochterski, R. L. Martin, K. Morokuma, V. G. Zakrzewski, G. A. Voth, P. Salvador, J. J. Dannenberg, S. Dapprich, A. D. Daniels, O. Farkas, J. B. Foresman, J. V. Ortiz, J. Cioslowski, D. J. Fox, Wallingford CT, **2009**.
[16] J. P. Perdew, K. Burke, M. Ernzerhof, *Phys. Rev. Lett.* **1996**, *77*, 3865.
[17] F. Weigend, M. Häser, H. Patzelt, R. Ahlrichs, *Chem. Phys. Lett.* **1998**, *294*, 143.
[18] V. Barone, M. Cossi, *J. Phys. Chem. A* **1998**, *102*, 1995.
[19] a) D. Andrae, U. Häussermann, M. Dolg, H. Stoll, H. Preuss, *Theo. Chim. Acta.* **1990**, *77*, 123; b) F. Weigend, R. Ahlrichs, *Phys. Chem. Chem. Phys.* **2005**, *7*, 3297.
[20] B. Metz, H. Stoll, M. Dolg, *J. Chem. Phys.* **2000**, *113*, 2563.
[21] a) F. L. Hirshfeld, *Theo. Chim. Acta.* **1977**, *44*, 129; b) A. E. Reed, R. B. Weinstock, F. J. Weinhold, *Chem. Phys.* **1985**, *83*, 735.
[22] Jmol, Jmol: an open-source Java viewer for chemical structures in 3D. http://www.jmol.org/
[23] G. Knizia, J. E. M. N. Klein, *Angew. Chem. Int. Ed.* **2015**, *54*, 5518; *Angew. Chem.* **2015**, *127*, 5609.
[24] OriginPro, OriginLabCorp., Northampton, MA, **2018**.
[25] M. L. Laury, M. J. Carlson, A. K. Wilson, *J. Comput. Chem.* **2012**, *33*, 2380.

[26] H. G. Von Schnering, M. Baitinger, U. Bolle, W. Carrillo-Cabrera, J. Curda, Y. Grin, F. Heinemann, J. Llanos, K. Peters, A. Schmeding, M. Somer, *Z. Anorg. Allg. Chem.* **1997**, *623*, 1037.

[27] WinXPOW, STOE&CieGmbH, Darmstadt, Germany, **2011**.

[28] a) D. Jianjun, F. S. Otto, *J. Phys.: Condens. Matter* **1999**, *11*, 6129; b) J. Dong, O. F. Sankey, G. K. Ramachandran, P. F. McMillan, *J. Appl. Phys.* **2000**, *87*, 7726; c) G. S. Nolas, C. A. Kendziora, *Phys. Rev. B* **2000**, *62*, 7157.

[29] a) F. Kiefer, V. Hlukhyy, A. J. Karttunen, T. F. Fässler, C. Gold, E.-W. Scheidt, W. Scherer, J. Nylen, U. Häussermann, *J. Mater. Chem.* **2010**, *20*, 1780; b) E. López-Cruz, M. Cardona, *Solid State Commun.* **1983**, *45*, 787.

[30] G. Kliche, H. G. von Schnering, M. Schwarz, *Z. Anorg. Allg. Chem.* **1992**, *608*, 131.

[31] C. W. Myles, J. Dong, O. F. Sankey, C. A. Kendziora, G. S. Nolas, *Phys. Rev. B* **2002**, *65*, 235208-1.

[32] a) H. Olijnyk, *Phys. Rev. B* **1992**, 6589; b) A. Göbel, J. Zegenhagen, M. Cardona, *Phys. Rev. B* **1997**, *56*, 13167.

[33] P. Franke, D. Neuschütz, in *Binary Systems. Part 5: Binary Systems Supplement 1: Phase Diagrams, Phase Transition Data, Integral and Partial Quantities of Alloys* (Eds.: P. Franke, D. Neuschütz), Springer Berlin Heidelberg, Berlin, Heidelberg, **2007**, pp. 1-4.
